# Anchored in the eye of the storm: a qualitative study of resilient performance during the COVID-19 pandemic in Sweden in the context of the emergency department

**DOI:** 10.1136/bmjopen-2024-094591

**Published:** 2025-03-03

**Authors:** Ann-Sofie Källberg, Camilla Göras, Lena Berg, Petronella Bjurling-Sjöberg

**Affiliations:** 1 Dalarna University School of Health and Welfare, Falun, Sweden; 2 Department of Emergency Medicine, Falun Hospital, Falun, Sweden; 3 Center for Clinical Research Dalarna, Falun, Sweden; 4 Department of Caring Sciences, Department of Occupational Health and Psychology, University of Gavle, Gavle, Sweden; 5 Department of Anesthesia and Intensive Care, Falun Hospital, Falun, Sweden; 6 Centre for Clinical Research Sörmland, Uppsala University, Uppsala, Sweden; 7 Department of Public Health and Caring Sciences, Uppsala University, Uppsala, Sweden; 8 Department of Patient Safety, Region Sörmland, Nykoping, Sweden

**Keywords:** COVID-19, Emergency Service, Hospital, Safety

## Abstract

**Abstract:**

**Objectives:**

This study aimed to explore how emergency department (ED) organisations and clinicians adapted to altered prerequisites during the first wave of the COVID-19 pandemic, the processes involved and the consequences. In addition, we examined how the ordinary state affected resilient performance during this period.

**Design:**

This qualitative study involved inductive thematic analysis of semi-structured interviews and narratives.

**Setting:**

Three hospital-based EDs, one county and two rural hospitals, located in two Swedish regions were studied.

**Participants:**

A total of 12 participants, 80% of whom were women, were recruited. The participants included two physicians, three registered nurses, three assistant nurses and four nursing managers working at the three EDs before and during the first pandemic wave.

**Results:**

The overarching theme ‘anchored in the eye of the storm’ emerged. This theme suggests that resilient performance during the pandemic was facilitated by ordinary adaptive capacity in the ED. A thematic map and seven main themes with a total of 25 subthemes explain the process. The ordinary state of conditions in the ED was challenged with the emergence of the COVID-19 pandemic. Altered prerequisites were perceived partly as a new reality in addition to business as usual. The adaptations included organise to regain control and developing new strategies to manage the situation, mainly by initiatives among clinicians. The consequences included perceived strain and frustration and partially impacted quality of care. However, an increased sense of cohesion among clinicians and enhanced knowledge were also noted.

**Conclusion:**

During the pandemic, a partially new reality was experienced, although work also continued largely as business as usual based on experiences of crowding, established preparedness plans and ordinary adaptive capacity. Despite dealing with a previously unknown patient group, the experience of working with critically ill patients and establishing structured work processes proved advantageous and facilitated resilient performance.

**STRENGTHS AND LIMITATIONS OF THIS STUDY**

- The study design provides new theoretical insights from empirical data, forming a basis for future research on resilient performance and aiding in managing current and future healthcare challenges.
- Participants from three emergency departments (EDs) in two Swedish regions, representing various professions, provided multiple perspectives on the same phenomenon.
- Recall bias was minimised by collecting data close in time after the first wave of the COVID-19 pandemic subsided.
- The research group included both experienced ED researchers and those without such experience to balance preconceptions with openness.
- While the limited number of departments included must be considered, transferability to other EDs is likely due to their generally similar working conditions and processes.

## Introduction

 The COVID-19 pandemic presented an immense challenge for society, especially for healthcare and, in particular, emergency departments (EDs).^1^ EDs and intensive care units were among the first hospital units to reorganise to manage the inflow of sometimes critically ill patients with suspected or confirmed COVID-19.^2 3^

Hospital EDs share similar organisational structures, workflows and challenges, both internationally and nationally.^4^ From a patient safety perspective, they are considered high-risk environments. They are characterised by dynamic networks of interconnected actions that cannot always be predicted because individual actions change the context for other individuals. These systems are often referred to as complex adaptive systems (CAS).^5^ A CAS has a self-organising nature that enables the system to remain stable despite challenges. However, changing dynamics also has the potential to destabilise the system.^6 7^ Over the past 20 years, intensive research has focused on how to identify and prevent patient safety risks in healthcare, including in EDs, to reduce harm.^8^,^10^ Recently, the focus on success factors for safety has increased.^11^ Patient safety is a multifactorial and critical aspect of healthcare. Resilience, defined as ‘the capacity to adapt to challenges and changes at different system levels, to maintain high-quality care’,^12^ is pivotal. Resilience requires an organisation to have the ‘potential’ for resilient performance, that is, the capacity to act in specific ways under certain conditions.^13^ However, the successful promotion of expedient resilient performance is still not fully understood.^14^

In ordinary situations, EDs face uneven and unplanned patient inflows, including patients with varying reasons for seeking care and severity levels. Patients are cared for over relatively short periods, often with the goal of keeping the total length of stay under 4 hours.^15^ Patients may be treated on-site or transferred to inpatient care. Consequently, emergency care has shifted from minor procedures and investigations to life-saving interventions. As a result of the unpredictable patient flow and varying levels of severity, crowding, defined as ‘when the identified need for emergency services exceeds available resources for patient care in the emergency department, hospital, or both’,^16^ occurs often^17^ and poses a significant challenge for most EDs worldwide.^18^

To manage the patient inflow and prioritise patients in need of urgent care, clinicians who work in EDs use structured assessment tools such as triage. Triage involves an initial assessment, most often conducted by a registered nurse (RN), of a patient’s level of urgency on the basis of symptoms and vital signs.^19^ There are various triage scales in use internationally, but most are divided into five levels, where level 1 represents the most urgent cases (patients needing immediate medical attention).^20^ The initial assessment of severely ill patients includes the structured and widely accepted Airway, Breathing, Circulation, Disability, Exposure approach.^21^ Following triage and subsequent physician evaluation, patient conditions are continuously monitored and re-evaluated via tools such as the National Early Warning Score to detect early signs of deterioration^22^ in an ongoing process. EDs also have other structured action plans, such as handling cardiopulmonary resuscitation^23^ and managing different types of trauma via Advanced Trauma Life Support.^24^ In addition to these standardised methods, EDs have well-established and regulated surge capacity preparedness plans.^25^,^28^ These plans include clear leadership structures, role assignments and action plans for temporary scenarios. During periods of crowding, the disaster preparedness structure, led by a physician or RN in charge, is used to allocate resources efficiently when scaling up.^29^

ED clinicians of all professions (physicians, RNs and assistant nurses) are accustomed to working conditions that include time pressure, interruptions and multitasking.^30^ Furthermore, ED clinicians are accustomed to responding to various situations, scaling up resources, reorganising and then returning to a calmer state. This adaptive capacity, known as resilience,^31^ is considered a core competence in emergency care.^32 33^

EDs were among the first hospital units that needed to respond to and reorganise their operations to handle the inflow of sometimes critically ill COVID-19 patients. This phenomenon enables us to draw lessons for future pandemics or other unpredicted long-term events. To our knowledge, previous studies have not examined the potential advantages of EDs’ pre-existing preparedness and working conditions and whether challenges arose despite this preparedness. The overall aim of the present study was to explore how EDs and clinicians adapted to altered prerequisites during the first wave of the pandemic, the processes involved and the consequences. An additional aim was to understand how ordinary conditions affected resilient performance during this period.

## Methods

The study was a part of the grounded theory research project ‘Resilient Performance in Healthcare during the COVID-19 Pandemic (ResCOV)’, which had the overall aim of better understanding the processes involved and the consequences.^34^ A qualitative explorative study design using thematic analysis was adopted.^35^

### Setting

The study was conducted in three hospital-based EDs in Sweden in two different regions. One ED was located at a county hospital, and two EDs were located at rural hospitals. The two regions had similar populations: each had approximately 300 000 residents and approximately 200 hospital beds per 100 000 inhabitants. In 2019, the county hospital ED and the two rural EDs had annual adult patient flows of approximately 38 000, 19 000 and 15 000, respectively.^36^

The EDs had similar specialties, including internal medicine, cardiology, surgical, orthopaedic, neurological and infectious disease experts. ED clinicians comprise physicians, RNs and assistant nurses. Physicians in the ED are traditionally consultants, residents and junior doctors from different specialties who are scheduled on an on-call basis. RNs are responsible for nursing care and medical-technical tasks, and they prepare and administer all prescribed medication. Assistant nurses have a high school degree and provide basic patient care under the supervision of RNs. Their duties typically also include monitoring vital signs and assisting with medical procedures.

### Participants and data collection

Participants were recruited from clinicians in the included EDs. A request, including information about the study, data collection procedures and details on voluntary participation, was sent to eligible clinicians via email. The information stated that the participants had the option to provide a written narrative or participate in a face-to-face interview, or do both, based on personal preferences. Purposive sampling was applied, aiming to capture a wide range of perspectives. This entailed the inclusion of all consenting clinicians until a satisfying variation of professions was ensured and data saturation was attained. A total of 12 participants, 80% of whom were women, were recruited. All participants completed a questionnaire that requested their demographic data. The participants consisted of two physicians, three RNs, three assistant nurses and four nursing managers working at the three EDs before and during the pandemic. The participants were between 29 and 62 years old. Their professional experience ranged from 5 to 30 years.

Data was collected during the last quarter of 2020 and included ten interviews and two written narratives. These data collection methods allowed us to capture deep and reflective information regarding the phenomenon under study, providing rich data to the analysis. Individuals who participated in interviews signed informed consent, and for participants who provided narratives, consent was assumed when their response was returned to the researchers. To facilitate interviews as well as the participants’ written narratives, a semi-structured guide was provided and developed in the ResCOV project.^34^ The guide included questions of interest for the study, such as working conditions and factors that affected them, patient safety, ethics, adaptations, consequences and lessons learnt. It started with an open question about how the participants experienced the first wave of the pandemic. Subsequently, clarifying and probing questions related to the topics of interest were asked (online supplemental file—Guide for Interviews and Narratives). The participants were also encouraged to include or exclude any information they considered relevant.

The research group in the present study consisted of four researchers, all female RNs and PhDs with extensive clinical experience in emergency care (A-SK and LB), intensive care (PB-S) and anaesthesia care (CG) as well as expertise in qualitative research. The researchers’ clinical experience facilitated communication with the participants and understanding of the context, and reflectivity was used to embrace preconceptions. Three of the researchers (A-SK, PB-S, CG) conducted interviews. A-SK was a former coworker in one of the EDs, but the other researchers did not have any established relationship with the EDs prior to the study commencement.

The interviews were conducted at a time and place chosen by the participants, mainly during their working time in a quiet room at the hospital. Each interview lasted between 21 and 72 min and was audio recorded and transcribed verbatim. It was determined that further or repeated interviews were not required, as it was perceived that saturation had been achieved.

### Data analysis

The data were analysed using thematic analysis with reference to Braun and Clarke and their six phases.^35^ The purpose was to identify themes in the data related to the study aim. We used an inductive process,^35^ which means that we let the data guide the coding without searching for predetermined categories. Throughout the process, strategies were employed to enhance trustworthiness in the study, which is further discussed in the Discussion section of the paper.

One of the researchers (A-SK) began by reading and rereading all transcribed interviews and narratives multiple times. Initially, this was done to become familiar with the material; later, this process was used to identify preliminary codes. Preliminary codes were extracted, labelled and organised using Microsoft Excel software (V.2412). Subsequently, researcher number two (LB) read through the data. The preliminary codes were discussed, compared and organised into different clusters and subthemes and preliminary themes began to crystallise. At this stage also the other two researchers (CG and P-BS) were involved.

All four researchers met multiple times to review, discuss, refine and structure the theme hierarchy in agreement. Table 1 provides examples of clustered codes depicting the subtheme, main theme, related domain and overarching theme. To visualise the final themes in a logical, consistent and interesting way, and to connect them with the overarching theme, we explored various models. Finally, a thematic map, which included the subthemes and main themes divided into three domains: prerequisites, adaptations and consequences.

**Table 1:** Examples of clustered codes depicting the subtheme, main theme, related domain and overarching theme

Clustered codes	Subthemes	Main themes	Domains	Overarching theme
Unclear, sometimes contradictory directives, which changed quicklyFear of the diseaseAccess to and use of protective equipmentUnknown patient group	Uncertainty about the situation	Partly new reality characterised by uncertainty and inadequacy	Prerequisites	Anchored in the eye of the storm

### Patient and public involvement

None.

## Results

From the interviews and narratives, an overarching theme emerged: ‘anchored in the eye of the storm’. Additionally, seven main themes were identified: *partly new reality*, *business as usual*, *organise to regain control*, *developing new strategies*, *strain and frustration*, *partially impacted quality of care* and *increased sense of cohesion and knowledge*.

**Figure 1:**
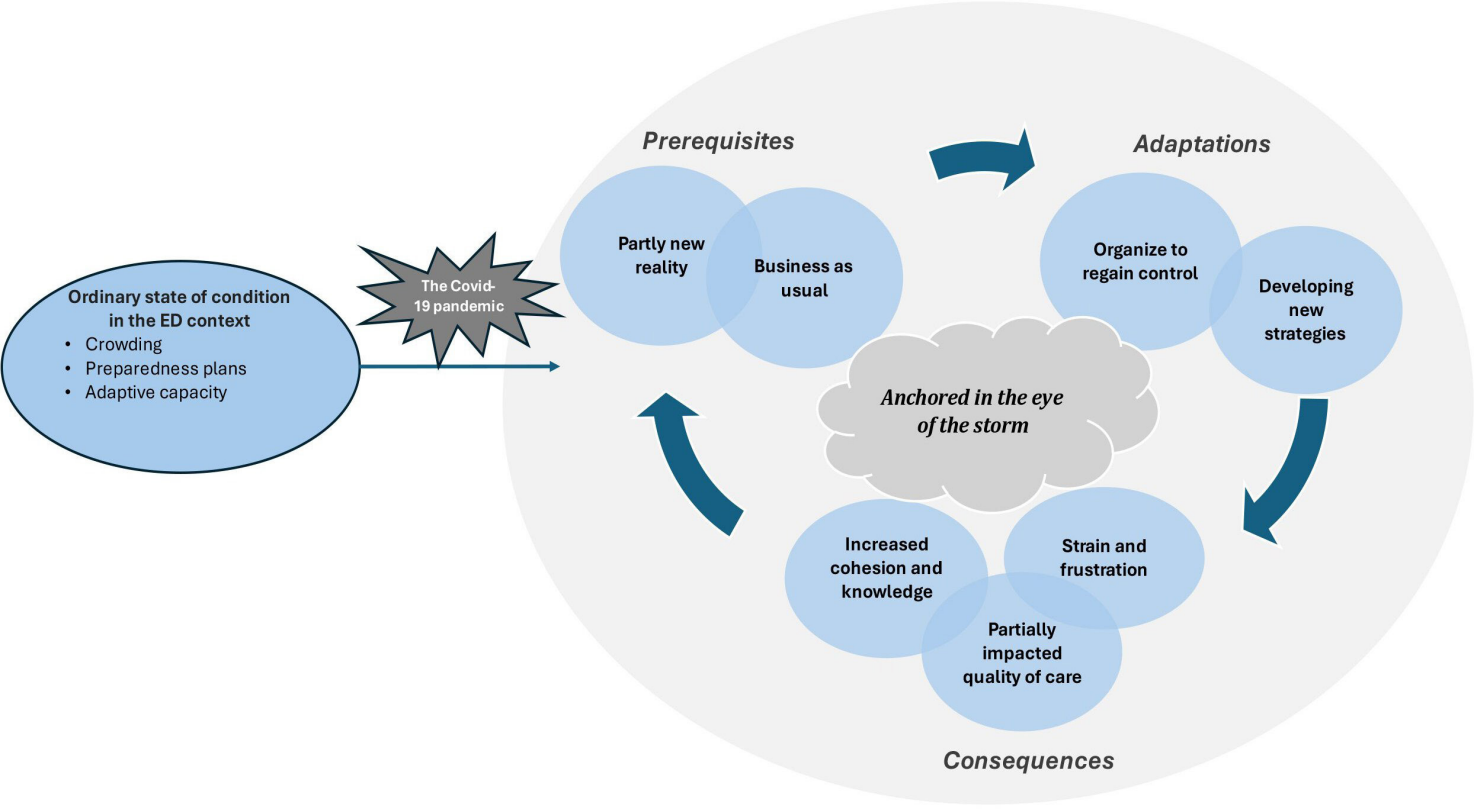
Thematic map of the process of resilient performance in the emergency department (ED), including the overarching theme, domains and main themes. The cycle of prerequisites and adaptations is visualised followed by various consequences, which lead to new altered conditions and subsequent adaptations.

These themes were associated with 25 subthemes (see table 2). The results are presented in the form of a conceptual model to illustrate how these different themes interact with each other (see figure 1). The findings are organised into three domains: the work environment *prerequisites* that ED clinicians had to consider during the first wave of the COVID-19 pandemic, the *adaptations* they made to address the altered prerequisites and the resulting *consequences* for both ED clinicians and patients.

**Table 2:** Overarching themes, domains, and main themes and subthemes that emerged from the interviews and narratives

Overarching theme	Anchored in the eye of the storm						
Domains	Prerequisites		Adaptions		Consequences		
Main themes	Partly new reality	Business as usual	Organise to regain control	Developing new strategies	Strain and frustration	Partially impacted quality of care	Increased sense of cohesion and knowledge
Subthemes	Different case mix of patientsUncertainty about the situationInadequate premisesOperational support	Established preparednessEstablished leadership rolesExperience with critically ill patientsAccustomed to being at the frontlineCommitted workforce	Revising and establishing new procedures and information channelsReorganising the premisesSecuring skill mix within the teams	Preparing for upcoming situationsTaking individual initiatives to find solutionsCreating safeguards to protect oneself	Frustration from difficulty and uncertainty regarding working conditionsPsychosocial impact on well-beingEthical stressStretching their own capacity limits	Concern about patient safety risksPrioritising patients with COVID-19 over othersAbsence of close relatives	Increased team spiritGained knowledge of managing the surge of contagious patients

### Prerequisites

The work environment prerequisites included the main themes: a *partly new reality*, and *business as usual*, which simultaneously existed in the EDs.

#### Partly new reality

When EDs were affected by the first wave of the COVID-19 pandemic, a *partly new reality* characterised by temporary uncertainty and increased inadequacy emerged for ED clinicians. This partly new reality consisted of a *different case mix of patients*, *uncertainty about the situation*, *inadequate premises* and *operational support* from other departments (table 2).

The new, *different case mix of patients* consisted of patient groups who normally presented to the ED and many patients with symptoms of COVID-19. The transition from a normal state to dealing with a large inflow of patients with signs of COVID-19 was described by the informants as rapid, and there was little time for the ED clinicians to prepare before this storm hit. The ED clinicians had to promptly comply with a variety of new guidelines, including how to sort and isolate patients with or without signs of COVID-19. Furthermore, they had to consider how to protect themselves from contracting COVID-19, symptoms and treatment strategies for COVID-19, and the introduction of restraint orders for close relatives at hospitals. Many of the new guidelines were unclear or even contradictory and changed rapidly, sometimes during a single shift. The informants described this as creating *uncertainty about the situation*. An assistant nurse described this experience:

It could be new routines … There could be one routine in the morning, and then it would have changed after lunch, and then a third one came before you went home. It was very chaotic. (AN 6)

Two areas that created particularly great uncertainty for the ED clinicians were the COVID-19 disease itself and the unknown patient group. Everyone initially had limited knowledge about how contagious COVID-19 was and how to treat it in combination with uncertainty about how ED clinicians should protect themselves from becoming infected. The informants noted that there were constantly new and sometimes even conflicting policies about what protective equipment to use in different situations and what guidelines they should adopt. This initially led to the inconsistent use of basic hygiene routines among clinicians.

At times, there was also great uncertainty about the availability of the right protective equipment. One manager remarked,

The working conditions were quite tough, as there was so much contemplation and worry and fear before we received any clear instructions on how to handle all the protective equipment. The directives were a bit back and forth, our staff felt. (M 2)

A major change in the partially new reality was the need to sort the different patient groups and separate patients with and without signs of COVID-19. The EDs solved this in different ways in relation to their local conditions, but the informants all described major challenges because of *inadequate premises* in the EDs. The premises were simply not designed and lacked space to effectively handle the sorting of such large numbers of patients who presented to EDs, as described by one RN:

The facilities … the deficiency I see is that there are not the basic prerequisites to have two streams of entry, one with a risk of infection and one for the others. (RN 7)

To manage the large inflow of patients and to allocate staff between patients with or without signs of COVID-19, *operational support* from various departments was required. This support included reallocating clinicians from other departments to the EDs as well as support from regional crisis management units, departments of infectious diseases, service and property departments and hospital churches. The informants described both the advantages and disadvantages of an increased workforce with inexperienced staff from other departments. More work labour was a positive aspect, but it sometimes created a greater workload for regular clinicians as new clinicians were not used to working in the ED context or dealing with emergency situations.

#### Business as usual

Although the partially new reality involved both uncertainty and changed working conditions for the clinicians, the informants also stated that work largely continued with *business as usual*. This was partially because of *established preparedness* and *established leadership roles*, both of which were part of the normal working conditions. As one assistant nurse expressed,

Being accustomed to rapid changes in procedures is something we’re quite familiar with in the emergency department, so I believe that it has been one of the factors contributing to our success. (AN 2)

Additionally, many patients with COVID-19 symptoms were critically ill, but the informants noted that they already had extensive *experience with critically ill patients* and handled these patients daily. One RN discussed new methods to assess patients with COVID-19:

This is a reassurance for me. Just like I use the NEWS to identify critical patients, I kneel a suspected COVID patient to identify the critical ones, so to speak. For me, it’s just another, not a vital parameter, but a test to ensure we can safely send them home. (RN 12)

Furthermore, several informants described the ED as the hub for questions and noted that other departments within the hospital asked them how to manage patients with COVID-19. ED clinicians are *accustomed to being on the front line* of hospitals. The ED clinicians also consisted of a *committed workforce*. The informants described a strong sense of loyalty, not only to each other but also to the organisation. They often volunteered for overtime, as one manager expressed:

Ending up in that part (major event) was not a challenge for the emergency department; on the contrary, the emergency staff work best then because we know what we have to deal with, and we work based on the conditions we have. (M 10)

They also stated that there is a certain type of person who chooses to work in the ED. These people appreciate when unexpected situations arise at work and, to some extent, find the new reality exciting.

### Adaptations

The prerequisites in the EDs meant that adaptations were needed. These adaptations appeared in the main themes *organise to regain control* and *developing new strategies*.

#### Organise to regain control

To cope with the altered prerequisites and the partially new reality, ED clinicians needed to adapt to respond to changes and *organise to regain control* of the situation. Adaptations meant that they repeatedly revised and *established new procedures and information channels*, *reorganised the premises* and *secured a skill mix within the teams* following new or altered directives. ED clinicians also volunteered often to work extra shifts to solve the staffing situation and had to find their own ways of coping. To manage the flow of constantly updated information on the management of patients with signs of COVID-19*, new procedures and information channels* needed to be revised and established. An example given by the informants was that meetings with hospital management, infection disease physicians and ED clinicians were held several times a day to ensure that they had constant access to updated information. One nurse manager remarked,

What should we use and what should we not use, and why should we listen to the infection control unit here and not listen to the public health agency and all the directives that come? There were discussions. (M 1)

However, preparedness at a higher level was uncommon, as a physician expressed:

The experience of how the organization responded is certainly not unique in any way, but it is not my task to draw those conclusions. The weaknesses that already existed became painfully clear; they could no longer be denied. (P 11)

As previously described, ED clinicians used their experience with business as usual, which formed the basis for making adaptations. These experiences also influenced the extent of the adaptations required. Furthermore, the informants reported that they largely kept working as usual but changed and scaled up certain aspects. As one manager explained,

We in the emergency department probably had an easier part, if I may say so, because we are used to working under direction and leadership that someone else decides. We sometimes have larger events where we have charge nurses where someone gives clear directives. (M 10)

To separate patients with COVID-19 symptoms from those without symptoms, the need to *reorganise the premises* in the ED emerged. One nurse manager reflected,

There was patient triage, you could say quite similar to usual, but there was an additional place for triaging patients. There were two lanes instead of one, as we had before. However, the staff quickly adapted. (M1)

Different solutions were used, such as triage tents outside the ED. The informants noted that specific clinicians took command to implement this reorganisation. Several of these clinicians had already established leadership roles in their day-to-day work during normal working conditions, such as charge nurses. This clear leadership allowed ED clinicians to quickly reorganise and find new solutions to control the situation. To *secure the skill mix within the teams*, rapid introductions of clinicians from other units at the hospital were introduced. One manager expressed the possible risk of this:

Well, we had maybe one or two nurses from the staffing center. But we saw that this posed a risk because they didn’t have the same experience as our nurses in properly assessing patients. So, I really had to be on my toes there. Ideally, I would have preferred to have our own nurses out there. (M 2)

#### Developing new strategies

The informants noted that the individual clinician took great personal responsibility for being able to work safely and maintain patient safety in the new conditions by *developing new strategies*. Examples of *developing new strategies* included *preparing for upcoming situations*, *taking individual initiatives to find solutions *and *creating safeguards to protect oneself*, as one physician described:

It was hot all over Sweden, but the solution was to tape overhead projector sheets. The county council management seemed quite pleased with this initiative. It was as if they wanted to showcase how much they were doing and how well prepared they were with these overhead visors. (P 11)

They made their own protective equipment when a shortage arose, such as a visor. They *prepared for upcoming situations*; for example, in their spare time, they created routines for the next shift and sought information independently about new findings about COVID-19. This creativity and *taking individual initiative to find solutions* continued over time as new ideas emerged, as expressed by an assistant nurse:

We only started about a month ago to put a blue note on all rooms with COVID and suspected COVID cases. We should have done that from the beginning, so it would have been clearer that we have COVID there. (AN 6)

They also chose to deviate from routines related to the presence of close relatives when they considered these deviations to be in the best interest of the patient, such as if there was a need for the relative to act as an interpreter or for bedside surveillance. As one assistant nurse remarked,

For a while, it was just ‘no’ to that. But it has become more flexible now. If it is assessed that this person needs to have their relative with them, they are allowed to come in. (AN 6)

### Consequences

Both negative and positive consequences arose as a result of the partly new reality and the adaptations that were made to handle the situation. These consequences affected both ED clinicians and patients as illuminated in the three main themes: *strain and frustration*, a *partially impacted quality of care* and an *increased sense of cohesion and knowledge*.

#### Strain and frustration

The ED clinicians were exposed to *strain and frustration*, which were evident in *frustration with difficult and uncertain working conditions*, *psychosocial impact on well-being* and *ethical stress*. This resulted in them *stretching their own capacity limits,* which negatively affected the ED clinicians. The informants gave several examples of *frustration from difficult and uncertain working conditions*, such as the extremely time-consuming and physically exhausting processes of putting on and taking off protective equipment and disinfecting all materials:

The major impact was all the protective clothing that always had to be worn and how difficult it is to talk to a patient in an empathetic and normal way when you’re wearing a mask, visor, and plastic clothing. (P 11)

It was also resource-intensive to work in parallel processes with COVID-19 patients and the regular patient flow. The participants gave several examples of the *psychosocial impact on well-being*, which included significant concerns about becoming infected with COVID-19 or infecting their family and having to spend much of their time away from their family due to the need to work extra hours. All of this contributed to feelings of exhaustion:

A lot of overtime. I can say that this has really worn down the staff. And the uncertainty, I think. Mentally. It drains the energy. And what we are thinking about for the next round now, which seems to be on the rise. (M 1)

Another negative consequence highlighted by the informants was the *ethical stress* they experienced towards patients and their families. They described a feeling of inadequacy, difficulty seeing patients with COVID-19 so critically ill and being unable to use their usual strategies to treat the symptoms, and patients sometimes dying without close relatives present:

It was really tough when severely ill COVID patients went directly to the ICU and were intubated in the emergency room, and you thought, ‘Will this patient survive?’ Someone had to call the relatives. And that’s not something we’re used to, even though we encounter a lot of sorrow and such. (M 2)

#### Partially impacted quality of care

For the patients, the informants described the feeling of *partially impacted quality of care*. They described *concerns about patient safety risks*, the need to *prioritise patients with COVID-19 over others* and the *absence of close relatives*, which was a negative experience for patients. They wondered whether there were non-COVID-19-infected patients with similar symptoms who had been sorted into the COVID-19 group and had become infected at the ED. They also described this, for example, in terms of length of stay in the ED and access to in-hospital beds. The *absence of close relatives* was perceived as having both advantages and disadvantages. For ED clinicians, it was sometimes easier to work without taking close relatives into consideration, but they believed that it was a negative experience for patients.

#### Increased sense of cohesion and knowledge

In addition to the negative consequences, the ED clinicians also experienced an *increased sense of cohesion and knowledge* were positive consequences for ED clinicians. The informants described an *increased team spirit* that was better than ever as a result of feeling that they were taking on a major challenge together. They also perceived that they *gained knowledge on how to manage a surge of contagious patients*. The experience of handling COVID-19 patients during the first wave of the pandemic caused them to become good at basic hygiene routines and preparedness in working with the flow and routines for COVID-19 patients, which could be quickly initiated again if a new wave occurred:

I feel that we must utilize this newly acquired competence among the staff before it is forgotten. We need to encourage and channel the inherent strength that has been built up and exists within the staff. (P 11)

## Discussion

The results presented in this paper can be summarised in line with the overarching theme: *anchored in the eye of the storm*. When the pandemic struck, ED clinicians faced altered prerequisites. A partially new reality was experienced, whereas work continued largely as business as usual despite a previously unknown patient group in addition to great uncertainty and inadequate premises. The general organisational adaptations aimed to help staff gain control over the situation in the ED, but there were also numerous adaptations initiated by individual clinicians. This generated a range of consequences, such as working conditions that were experienced as strenuous and frustrating, which were perceived to contribute to partially reduced patient safety. However, there were also positive consequences, such as an increased sense of cohesion and team spirit as well as gaining new knowledge.

The present findings indicate that EDs in their ordinary state have adaptive capacity on the basis of established preparedness plans and experiences of crowding. ED clinicians were accustomed to working at the front lines and handling critically ill patients while managing crowding. Such conditions often result in multitasking, which is a key skill for ED clinicians^37^ but also a cause of stress.^38^ However, multitasking can increase situational awareness,^37^ which may have been an advantage during the pandemic. Situational awareness in an ED is required to manage rapid changes in workload, but at the same time, situational awareness is impaired by constant interruptions.^39^ EDs are known to be interruption-driven environments with consequences for patient safety.^30 40^ From our findings, it appears that established preparedness and awareness of situations allowed ED clinicians to quickly adapt both collectively and individually to the altered prerequisites. In addition to professional experience, established routines such as triage^4^ and disaster preparedness plans^26^ provided a foundation and the feeling of being anchored in the storm.

The adaptations made to gain control of the situation were largely conducted by the staff themselves. Their adaptability was largely due to strong initiative among the clinicians, partly because they were accustomed to solving problems quickly even under normal circumstances. The situation was managed largely by the clinicians’ strong sense of teamwork and their ability to stretch themselves to the limit, which is supported by Rasmussen’s safety theory.^41^ The results of our study confirm theories about system resilience, namely, that the ability to adapt largely exists within individuals in the system.^42^ Clinicians’ experience, strategies and tools contribute to adaptive capacity. However, this is not normative; expressions of resilient performance may also hide deficiencies higher in the healthcare system.^43^ At the frontline level, the objective of providing safe care while maintaining an acceptable workload can result in conflicting goals between individuals and the organisation with regard to patient safety and/or personal safety. When equilibrium is achieved between patient safety and workload, work can be accomplished within the safety margin defined by the organisation. However, it is challenging to ascertain the threshold at which a system or individual may be at risk of experiencing burnout or harm to patients. The COVID-19 pandemic provides an illustrative example of an unexpected event with considerable pressure on the healthcare system. It also demonstrated the limitations of what both systems and individuals can manage in the context of a crisis. Rasmussen’s safety model^41 44^ elucidates not only how the system can expand, for example, in response to crowding but also how individuals under pressure may reduce ‘unnecessary tasks’ to increase efficiency; this may be achieved at the expense of thoroughness, thereby jeopardising the safety of both patients and clinicians.^45^ Moreover, if the margins are gradually expanded without deviation, clinical practice and shared values may drift toward acceptance of unsafe behaviours and deviation from protocols, a phenomenon is known as the normalisation of deviance.^46^

The greatest challenge seemed to be separating patients with suspected COVID-19 from other patients. It was quickly discovered that EDs are not built for pandemics, as reported in other studies.^347^,^50^ This resulted in many relocations to tents, separate rooms, and temporary outdoor facilities. ED clinicians also tried to combine experienced staff with inexperienced staff to temporarily help them achieve a reliable competence mix. Individual solutions included marking doors to rooms of patients with suspected infection. Another major challenge appeared to be the shortage of protective equipment. Basic hygiene routines were put to the test and initially seemed to be lacking. Basic hygiene routines with hand sanitiser are the most effective methods to avoid infection. Unfortunately, compliance is sometimes very low. This is a global issue that the WHO has noted, and guidelines have been created.^51^ One reported reason for low compliance could be crowded EDs,^52^ which was the case during the pandemic. The fear of becoming infected or infecting one’s relatives complicated the situation, as previously reported.^53^

The present findings indicate that resilient performance was achieved because of ED clinicians’ adaptability,^54^,^56^ established preparedness^49^ and situational awareness,^57^ demonstrating the importance of emergency medicine as a pillar during the pandemic.^58^ This was further supported by a committed workforce with structured routines and working methods coupled with experience managing crowding. What set this pandemic apart from other unexpected events is that it was prolonged, which pushed the staff to their limits. However, since ED clinicians already had inherent situational awareness and knew that working conditions could change quickly, they did not seem to have much difficulty adapting early in the first wave. This contrasts with experiences from ICUs, where the initial phase of the pandemic was perceived as chaotic because of the unforeseen and sudden need to adapt to the situation.^2^

The consequences during and after the pandemic that affected ED clinicians, patients and their relatives might, to some extent, be due to shortcomings at the level of both regional and national management. Pre-existing regional shortcomings were highlighted during the pandemic, particularly the inadequacy of premises in EDs and a shortage of experienced clinicians.^59^ Nationally, the lack of protective equipment and inconsistent and sometimes conflicting directives were subject to frequent changes. Notably, there was a lack of unified strategies for addressing pandemics despite the proximity of healthcare to previous pandemics and knowledge of the inevitability of such crises.^28 60^ The potential for more severe consequences, such as burnout, increased sick leave and patient safety risks, was mitigated by ED clinicians’ experience, preparedness and adaptability at the microlevel.^58 61^

The present study has both strengths and limitations that have to be considered. Strategies to ensure trustworthiness were employed according to thematic analysis^62^ and criteria for reporting qualitative research.^63^ Two of the researchers had extensive clinical experience working in EDs, which raised the concern that their preconceptions might influence the analysis. However, this experience, combined with qualitative research expertise, contributed to a deeper understanding of the phenomenon explored. To further improve credibility, an additional researcher reviewed the initial coding followed by separate individual analyses and subsequent comparisons. Furthermore, two researchers who did not have ED experience were involved in the final analysis to balance the risk of the analysis being affected by preconceptions. The participants were recruited from three EDs located in two different regions and came from various professions to provide multiple perspectives on the same phenomenon. The majority had extensive experience working in EDs both before and after the pandemic, which allowed them to compare how unexpected situations were handled during so-called normal circumstances versus more extreme situations such as the pandemic. Dependability was reinforced by using a semi-structured interview guide and conducting all data collection during the same period. Data collection was retrospective, which might have led to recall bias. However, data were collected when the first wave had subsided, but a second wave was imminent. This allowed the participants to reflect on the situation close to the first wave. Although they may have forgotten certain details, this contributed to avoiding recall bias.^64^ Transferability to other EDs is likely possible as they generally share similar ordinary working conditions and processes.

## Conclusion

During the first wave of the COVID-19 pandemic, EDs experienced a partially new reality, although work continued largely as business as usual on the basis of staff members’ experience and established preparedness plans. The adaptations included organising to regain control and developing new strategies to manage the situation. The resilient performance was largely due to strong initiative among the clinicians, who were accustomed to solving problems quickly even under normal circumstances. Despite dealing with a previously unknown patient group, the experience of working with critically ill patients and established, structured work processes seemed to be advantageous. This study provides a novel theoretical understanding based on empirical data. It can serve as an important basis for future studies of organisational and individual resilience and the ability to manage current and future challenges in healthcare. However, further research is needed on the importance of organisational and individual factors for achieving sustainability in the ED during prolonged crises such as pandemics.

## supplementary material

10.1136/bmjopen-2024-094591 online supplemental file 1

## Data Availability

Data are available upon reasonable request.
