# Supplementary material for: Anchored in the eye of the storm: a qualitative study of resilient performance during the COVID-19 pandemic in Sweden in the context of the emergency department
Source: BMJ Open. 2025 Mar 3;15(3):e094591. doi: 10.1136/bmjopen-2024-094591 (PMC11877203; doi:10.1136/bmjopen-2024-094591)
Supplement: Supplementary file 2 [file bmjopen-2024-094591.reviewer_comments.pdf]

PEER REVIEW HISTORY

BMJ Open publishes all reviews undertaken for accepted manuscripts. Reviewers are asked to complete a checklist review form and are provided with free text boxes to elaborate on their assessment. These free text comments are reproduced below.

ARTICLE DETAILS

Title (Provisional)

Anchored in the Eye of the Storm: A Qualitative Study of Resilient Performance during the COVID-19 Pandemic in Sweden in the Context of the Emergency Department

Authors

Källberg, Ann-Sofie; Göras, Camilla; Berg, Lena; Bjurling-Sjöberg, Petronella

---

VERSION 1 - REVIEW

**Reviewer**	**1**
**Name**	**Khazaei, Afshin**
**Affiliation**	**Asadabad School of Medical Sciences**
**Date**	**19-Oct-2024**
**COI**	**None**

Summary of Corrections and Suggestions

1. Clarify the roles, experiences, and demographics of the research team members.

2. Clearly indicate participant selection methods, approach, and sample characteristics.

3. Discuss how data collection methods impacted the results and theme development.

4. Increase transparency about the analysis techniques and tools used.

5. Continually relate participants' responses to overarching themes in the results.

Overall, the article is quite rigorous, but focusing on these areas will improve transparency, methodological rigor, and overall adherence to the COREQ criteria.

Dear Editor

I would like to extend my sincere gratitude for the opportunity to review the manuscript titled **Anchored in the Eye of the Storm: A Qualitative Study of Resilient Performance during the COVID-19 Pandemic in the Context of the Emergency Department**." I appreciate the trust you have placed in me to evaluate this work.

The manuscript is well-written and presents valuable insights; however, I have noted some areas that require further refinement. I have provided detailed comments and suggestions for improvement in my review, which I believe will enhance the clarity and overall quality of the manuscript.

Thank you once again for the opportunity to contribute to the review process. I look forward to seeing the revised version of the manuscript.

- Please include the demographic characteristics of the participants in the manuscript in the form of a table?
- Please attach the main questions and probes. State whether the guide was pre-tested and provide an example of questions used.
- How many people refused to participate or dropped out? Reasons? Add information regarding non-participation if applicable.
- Where was the data collected? e.g. home, clinic, workplace. Clarify the settings more explicitly
- Were repeat interviews carried out? If yes, how many? Clarify this for transparency purposes.
- Did the research use audio or visual recording to collect the data
- Identify which author(s) conducted the interviews. Please Clearly state the names of the interviewers at the beginning of the methodology section.
- Was a relationship established prior to study commencement? Not explicitly stated. Explain any prior relationships with participants to clarify potential bias.
- What were the researcher's credentials? E.g., PhD, MD? Include specific qualifications and roles of each researcher involved. Clearly summarize the authors' occupations in the methodology section. State the gender of each interviewer specifically.
- What experience or training did the researcher have? **: Relocate this information closer to the methodology section, specifying how it influenced the research.
- Were field notes made during and/or after the interview or focus group?
- Was data saturation discussed? Discuss the manner in which saturation was monitored or achieved.
- In the present study, there is no mention of trustworthiness? How did you do the rigor of the study? Please mention this in the text of the manuscript.
- How many data coders coded the data? Mentioned that multiple researchers coded data. Confirm exact number coding for precision.
- Did authors provide a description of the coding tree? If yes, please create a diagram or table summarizing the coding tree.
- What software was used? Include mention of coding software used in the analysis section (e.g., NVivo), if any.
- Did participants provide feedback on the findings? Discuss how this may affect study validity if applicable.

Reviewer **2**

Name **Skene, Imogen**

Affiliation **Wolfson Institute of Population Health, Centre for Primary Care and Mental Health**

**Date**	**16-Dec-2024**
**COI**	**None**

Dear Authors,

Thank you for the opportunity to review your paper on how Emergency Department staff adapted during the first wave of the pandemic.

This work is important to understand the impact of pandemic on Emergency Departments processes. I write my review as a nurse with experience of working the ED during this period. I found the results of your study spoke to me, reflecting the reality of my experience.

I have some minor comments which I hope you will find useful and constructive.

While you have noted that in Sweden, the doctors are traditionally from different specialities, rather than specialist ED doctors, it may be helpful to include their primary speciality/level of ED experience and to comment on if this difference was something to come out of the interviews (i.e compared to full time ED staff)? Were there difference between the professional groups that were highlighted in the results?

Please could you clarify the role of an assistant nurse, this isn't a role I am familiar with. Is it a regulated profession, or like our healthcare assistant roles in the UK?

Please provide a justification for stopping at 12 participants, was it data saturation or another criteria?

Clarify in the methods if potential participants were given the option of either/both an interview or written narrative? Was there a reason one was chosen over the other – personal preference, timing? Were the semi structured interview questions the same for the narrative report?

In the data analysis section, it would be helpful is researcher one/two could be indicated with the initials of the author in the published version.

In the discussion, short comings of higher management is brought up, it is unclear if this is directed a hospital management or at a national level? From the results, you have highlighted the challenges of the directives coming from different levels (ie public health or hospital infection control).

VERSION 1 - AUTHOR RESPONSE

Reviewer 1:

Summary of Corrections and Suggestions

1. **Clarify the roles, experiences, and demographics of the research team members.**

   Authors response:
   Thank you for the suggestions. We have revised the method section to include this information, based on the recommendations provided in the detailed comments below.

2. **Clearly indicate participant selection methods, approach, and sample characteristics.**

   Authors response:
   Thank you for highlighting our insufficient transparency. We have made some revisions in the method section to clarify participant selection methods, approach, and sample characteristics, as described in the detailed comments below.

3. **Discuss how data collection methods impacted the results and theme development.**

   Authors response:
   Thank you for the suggestion, we have added some sentences regarding this in the method section.

4. **Increase transparency about the analysis techniques and tools used.**

   Authors response:
   Thank you for making us aware that this was not clearly stated. We have revised the Data analysis section to increase transparency, as described in the detailed comments below.

5. **Continually relate participants' responses to overarching themes in the results.**

   Authors response:
   Thank you for illuminating that this was not clear in the manuscript. We have made some amendments in the result section to overcome this issue.

Additional details:

a. **Please include the demographic characteristics of the participants in the manuscript in the form of a table?**

   Authors response:
   Thank you for the suggestion. The demographic data of the participants is described in continuous text embedded in the method section. Since there is a small amount of data and few individuals, we think continuous text is more appropriate than a table. However, to facilitate for the reader to find the information we have made some adjustments of the subheadings and structure of the text. If you do not think this amendment is satisfying,

we are open to provide a table upon request.

b. **Please attach the main questions and probes. State whether the guide was pre-tested and provide an example of questions used.**

Authors response:
We have now provided a supplementary file including the guide used (translated to English). The guide was developed and tested in the ResCOV project, in which the present study is a part. We have now included this information in the manuscript.

How many people refused to participate or dropped out? Reasons? Add information regarding non-participation if applicable.

Authors response:
Thank you for making us aware that we have not been clear in this matter. We asked about participation in the study via email, stating that we were looking for individuals who were likely affected by the changes during the pandemic and therefore had experiences to share. We aimed for a variety of professional roles to obtain comprehensive data. After 12 participants we determined that received data were sufficient for analysis and that additional inclusion of participants would not have contributed further. No participants withdrew from the study, so no data are available on that subject. We have now revised parts of the method section to clarify the sampling process.

c. **Where was the data collected? e.g. home, clinic, workplace. Clarify the settings more explicitly**

Authors response:
We have added information about where data was collected.

d. **Were repeat interviews carried out? If yes, how many? Clarify this for transparency purposes.**

Authors response :
Thank you for highlighting our insufficient transparency. No repeated interviews were conducted. The information is added in the manuscript.

e. **Did the research use audio or visual recording to collect the data**

Authors v response:
We used audio recording and this information is now added to the manuscript.

f. **Identify which author(s) conducted the interviews. Please Clearly state the names of the interviewers at the beginning of the methodology section.**

Authors response :
We have now added information to clearly state which researchers that conducted the

interviews.

g. **Was a relationship established prior to study commencement? Not explicitly stated. Explain any prior relationships with participants to clarify potential bias.**

Authors response :
Thank you for highlighting our insufficient transparency. We have added information that one of the researchers had clinical experience from one of the EDs.

h. **What were the researcher's credentials? E.g., PhD, MD? Include specific qualifications and roles of each researcher involved. Clearly summarize the authors' occupations in the methodology section. State the gender of each interviewer specifically.**

Authors response :
We have added information in the method section, based on your recommendation.

i. **What experience or training did the researcher have? **: Relocate this information closer to the methodology section, specifying how it influenced the research.**

Authors response :
We have added some information and relocated some information previously placed in the Discussion section.

j. **Were field notes made during and/or after the interview or focus group?**

Authors response :
This is a legitimate question. We only performed individual interviews, which all were audio recorded. No fieldnotes were taken, thereby not mentioned in the manuscript. However, your question raised concerns that we have not been clear regarding the type of interviews performed. Thereby we have made som addmandments throughout the manuscript.

k. **Was data saturation discussed? Discuss the manner in which saturation was monitored or achieved.**

Authors response :
We determined that retrieved data were sufficient for analysis and that additional data collection would not have contributed further. Saturation was achieved. We have added this information.

l. In the present study, there is no mention of trustworthiness? How did you do the rigor of the study? Please mention this in the text of the manuscript.

Authors response :
Thank you for illuminating that readers might not find this important information in our paper. Trustworthiness and strategies employed are described and discussed in the

Discussion section. We have added some clarifying information in the initial sentence. Additionally, since readers have different preferences and are used to different journal styles we have now added a sentence in the method section to guide the readers

m. **How many data coders coded the data? Mentioned that multiple researchers coded data. Confirm exact number coding for precision.**

Authors response :
Thank you for highlighting our insufficient transparency. We have added a more detailed description of the analysis process and the roles of the different researchers.

n. **Did authors provide a description of the coding tree? If yes, please create a diagram or table summarizing the coding tree.**

Authors response :
During the analysis process, identified codes were organized into clusters and subsequently into thematic hierarchies, which successively resulted in the final thematic map. Example of the process is provided in Table 1. The thematic map is provided in Figure 1 and further described in detail in Table 2. We have made some modifications of the text to further clarify this.

o. **What software was used? Include mention of coding software used in the analysis section (e.g.,NVivo), if any.**

Authors response :
We did not use any specific coding software. However, Microsoft Excel was used to keep track of all codes. We have revised the information of this to further clarify

p. **Did participants provide feedback on the findings? Discuss how this may affect study validity if applicable.**

Authors response :
Feedback from participants can help identify eventual biases from the researchers' preconceptions that might have influenced the analysis, but we did not utilize this validation method. However, as described in the manuscript, collaboration in the analysis process and reflective discussions in the research group, which consisted of researchers with and without ED experience, contributed to improving credibility.

Reviewer 2:

- **While you have noted that in Sweden, the doctors are traditionally from different specialities, rather than specialist ED doctors, it may be helpful to include their primary speciality/level of ED experience and to comment on if this difference was something to come out of the interviews (i.e compared to full time ED staff)? Were there difference between the professional groups that were highlighted in the results?**

Authors response:
Thank you for raising an interesting question. We have added some information about the two doctors. However, our sample size in the present study did not allow for comparisons between groups.

- **Please could you clarify the role of an assistant nurse, this isn't a role I am familiar with. Is it a regulated profession, or like our healthcare assistant roles in the UK?**

  Authors response:
  Thank you for illuminating this issue. We are not familiar with the healthcare assistant role in the UK but have added information about the Swedish assistant nurses' education level and role.

- **Please provide a justification for stopping at 12 participants, was it data saturation or another criteria?**

  Authors response:
  Thank you for illuminating that we had not been clear in this matter. It was because of data saturation and this information has been added in the method section.

- **Clarify in the methods if potential participants were given the option of either/both an interview or written narrative? Was there a reason one was chosen over the other – personal preference, timing? Were the semi structured interview questions the same for the narrative report?**

  Authors response:
  The participants could choose between an interview, writing, or both, based on personal preferences. We have added this information in the manuscript. We do not have information for the reason of their choice. The question guide for narrative report included the same topics as for the interviews. We have modified some sentences in the method section to further illuminate this and also attached the guide as Supplementary material.

- **In the data analysis section, it would be helpful is researcher one/two could be indicated with the initials of the author in the published version.**

  Authors response:
  Thank you for the suggestion, we have added the initials

In the discussion, short comings of higher management is brought up, it is unclear if this is directed a hospital management or at a national level? From the results, you have highlighted the challenges of the directives coming from different levels (ie public health or hospital infection control).

Authors response:

Thank you for illuminating that we had not been clear in this matter. We have clarified that the short comings were at both regional and national management level.

VERSION 2 - REVIEW

**Reviewer**	**1**
**Name**	**Khazaei, Afshin**
**Affiliation**	**Asadabad School of Medical Sciences**
**Date**	**12-Feb-2025**
**COI**	

Thanks for the corrections. The manuscript is much better based on the Consolidated Criteria for Reporting Qualitative Research (COREQ) guidelines. Congratulations to the authors on their excellent manuscript.
